# Application of a Blockchain Platform to Manage and Secure Personal Genomic Data: A Case Study of LifeCODE.ai in China

**DOI:** 10.2196/13587

**Published:** 2019-09-10

**Authors:** Xiao-Ling Jin, Miao Zhang, Zhongyun Zhou, Xiaoyu Yu

**Affiliations:** 1 Management School, Shanghai University Shanghai China; 2 Department of Management Science and Engineering, School of Economics and Management, Tongji University Shanghai China; 3 SHU Center for Innovation and Entrepreneurship, Shanghai University Shanghai China

**Keywords:** genomics big data platform, blockchain, data ownership, data sharing, data security, digital health

## Abstract

**Background:**

The rapid development of genetic and genomic technologies, such as next-generation sequencing and genome editing, has made disease treatment much more precise and effective. The technologies’ value can only be realized by the aggregation and analysis of people’s genomic and health data. However, the collection and sharing of genomic data has many obstacles, including low data quality, information islands, tampering distortions, missing records, leaking of private data, and gray data transactions.

**Objective:**

This study aimed to prove that emerging blockchain technology provides a solution for the protection and management of sensitive personal genomic data because of its decentralization, traceability, encryption algorithms, and antitampering features.

**Methods:**

This paper describes the case of a blockchain-based genomic big data platform, LifeCODE.ai, to illustrate the means by which blockchain enables the storage and management of genomic data from the perspectives of data ownership, data sharing, and data security.

**Results:**

Blockchain opens up new avenues for dealing with data ownership, data sharing, and data security issues in genomic big data platforms and realizes the psychological empowerment of individuals in the platform.

**Conclusions:**

The blockchain platform provides new possibilities for the management and security of genetic data and can help realize the psychological empowerment of individuals in the process, and consequently, the effects of data self-governance, incentive-sharing, and security improvement can be achieved. However, there are still some problems in the blockchain that have not been solved, and which require continuous in-depth research and innovation in the future.

## Introduction

### Background

The rapid development of next-generation sequencing and genome editing has dramatically reduced the cost of single genome-wide testing. This technology has facilitated the global adoption and wide application of gene sequencing, a technique that once existed only in cutting-edge research laboratories, in medical and health care institutions such as hospitals and clinics around the world. Its rise reflects the increasing interest among both researchers and practitioners in the interdependence between gene data analysis and medical treatment. Moreover, it has been demonstrated that gene sequencing has many applications, such as enhancing chronic disease prediction, new drug development, and precision medicine [1-4].

Despite the benefits of gene sequencing, the storage, transmission, and management of genomic big data in a secure fashion presents a great challenge. This is a critical issue for the development of gene sequencing and has thus been of interest to both researchers and practitioners. To address this issue, an increasing number of companies and institutions are using advanced computational tools, including big data analysis and cloud computing, to construct genomics big data platforms to integrate personal genomics data [5,6]. These platforms offer support services to a variety of user groups (eg, patients, medical institutions, and insurance companies) in domains such as data archiving, computational analysis, knowledge search, management authorization, and association visualization, and they have been leveraged for chronic disease prediction, new drug research and development (R&D), and precision medicine [7].

### Challenges of Genomics Big Data Platforms

The core function of genomics big data platforms is to collect personal genetic data for application and sharing. However, the collection and sharing of genetic data is often challenged by problems such as low data quality, information islands, tampering distortions, missing records, leaking of individual privacy, and gray data transactions. The key challenges that hinder the development of genomics big data platforms can be classified into 3 facets: data ownership, data sharing mechanisms, and data security [8-11].

#### Unclear Ownership of Data

It is difficult to define data ownership due to its replicability and virtual nature. As no laws or regulations provide detailed guidelines, the ownership of personal health data remains controversial, and various stakeholders have different opinions on data ownership issues, especially in China. It is thus imperative to correctly determine the ownership of medical data.

#### Insufficient Data Sharing

Data can produce value with interoperability and sharing, and data analysis is an important way to promote the development of health care and medicine [12]. However, personal data owners are reluctant to share genomic data without effective sharing incentives and without a clear value exchange [13,14]. As a result, the benign self-running sharing mechanism design of the data platform has great importance.

#### Privacy Leakage and Insecurity

Human DNA contains extremely sensitive and private information [15], so individuals’ privacy may be jeopardized when genomic and medical data are spread or shared. Health data have traditionally been stored by a single organization in a centralized fashion, but the data may be taken without the users’ consent for unintended purposes, resulting in a threat to the data owners [16,17]. Hence, privacy protection and security are fundamental to the collection and application of genomic big data.

### Application of Blockchain

Blockchain is an emerging technology that has attracted increasing attention from both researchers and practitioners. Briefly, a blockchain is a publicly distributed ledger that seals blocks with timestamps and encrypted hash links in a secure and immutable manner [18,19]. It enables transaction processes without the need for a trusted third party because of its traceability, smart contracts, resistance to tampering, decentralization, and encryption algorithm [20]. First, a blockchain uses timestamp technology to achieve data traceability and verifiability, which means that it offers a secure and transparent method to track the ownership of assets before, during, and after any transaction [21]. Second, the open-source sharing protocol built into blockchain enables simultaneous data logging and storage by all participants, which ensures that the details of the recorded transactions cannot be retroactively changed without the full agreement of the network [22,23]. Finally, a blockchain’s architecture and governance are decentralized, which makes it highly fault-tolerant and resistant to attacks and collusion. Moreover, a blockchain can protect users’ privacy with the use of cryptographic hash functions and asymmetric encryption to enable users to encrypt data with their own private key [24-26].

As such, a blockchain provides a new method of solving the problems encountered in the construction of genomics big data platforms. Specifically, it can empower a genomics big data platform in at least 3 ways.

#### Traceability Empowers Data Ownership

Traceability is the ability to verify an item’s history, location, or application by means of documented recorded identification [27]. Blockchain uses timestamp technology to make data traceable and verifiable, so it completely records the entire process from data generation to final storage [28]. Thus, each piece of data on the blockchain can be determined to be owned by the individual data producer at the time of generation, and this data ownership is verifiable based on the record.

#### Antitampering and Smart Contracts Empower Data Sharing

A smart contract contains a set of rules that help the parties to the smart contract interact automatically [29]. No record on the blockchain can be tampered with by any individual node [29]. The smart contract code facilitates, verifies, and enforces the negotiation or performance of an agreement or transaction, and the antitampering feature helps implement access and protect the data’s originality, thus ensuring the operation of smart contracts [30-32]. Therefore, the application of these 2 characteristics of blockchain can improve the data sharing mechanism, which then stimulates the platform’s participants to share data for extrinsic benefits.

#### Decentralization and Encryption Algorithms Empower Data Security and Privacy Protection

Decentralized storage helps to reduce security risks, increase trust, and manage data [33]. An encryption algorithm is a component for electronic data transport security. The blockchain encryption algorithm encrypts the data and strongly guarantees data security and privacy [34]. For example, Sun et al [35] proposed the use of blockchain technology to protect intellectual property and data security and privacy in internet education.

### Objective and Research Questions

The research on blockchain in the field of health care focuses primarily on technical aspects such as the algorithm model, feasible solutions, and structural design. For example, Yue et al [36] designed an app architecture that enables patients to own, control, and share their data easily and securely without violating their privacy by using blockchain-based platform. Zhang et al [37] implemented a blockchain-based decentralized app (DApp) to address interoperability challenges in smart health care. On the basis of blockchain developed for telecare medical information systems, Ji et al [38] proposed a novel blockchain-based multilevel privacy-preserving location-sharing scheme to realize location sharing while preserving privacy. Few studies, however, have focused on revealing how to apply blockchain technology to empower genetic engineering. With this motivation, this study aims to address the following research question:

RQ: How does blockchain technology empower the storage and management of genomics data in a genomics big data platform?

To answer this question, we report a case study of a blockchain-based genomics big data platform in China, LifeCODE.ai, to explore the effectiveness of blockchain in the management and security of personal genomic data.

## Methods

### Case Study Method

To address the proposed research question, we used case study as the research method because of the following reasons. First, the application of a blockchain platform to manage and secure personal genomic data is a complicated process, the adoption process concerns the *how* question, and the case study method is suitable for a process-based analysis [39]. In addition, the case study research method has a practical nature and can increase the effectiveness of the evidence [40]. Finally, the case study method has a strong descriptive nature, conveys a clear analysis of events and a strong sense of reality, and is easily understood by readers [41-43].

Given the research question, the selection of a case was subject to certain conditions. First, the case organization needed to be a business in biomedical health care and/or medicine. Second, the case organization was required to apply a blockchain platform to address genetic data management and security issues. Third, the case organization had to be willing to accept repeated surveys.

**Figure 1:**
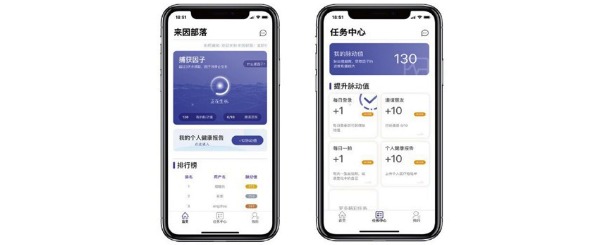
Laiyin Health app interface.

On the basis of these conditions, we selected LifeCODE.ai, a genomics big data platform based on blockchain technology, as the object of this study. LifeCODE.ai was first released at the TechCrunch International Innovation Summit 2018. It uses a decentralized consensus approach to construct a blockchain platform to aggregate distributed health data. With the ultimate goal of improving overall health outcomes via genomics research, LifeCODE.ai comprises a secure, decentralized, and visible personal health data center that removes the boundaries of health information between hospitals, pharmaceutical R&D institutions, doctors, patients, and individuals. At the same time, LifeCODE.ai released to individual users the blockchain-based DApp, Laiyin Health, which also applies blockchain for data self-governance implementation, token mechanism design, and encryption algorithm application. Figure 1 shows the interfacial design of this DApp. Unlike traditional apps whose backend code is run on centralized servers, the backend code of a DApp is run on a decentralized peer-to-peer network [44]. LifeCODE.ai is available on the official website [45].

On-site data were then collected via 2 interviews of the company that developed and manages LifeCODE.ai, its departmental managers were interviewed, and the operation of LifeCODE.ai and the application of blockchain in 2018 were discussed. The interviewees included corporate executives and department managers at LifeCODE.ai, and each interview lasted approximately 60 to 120 min. Each interview was digitally recorded and transcribed. At the same time, we systematically collected secondary data about this platform from a variety of sources, including newspapers, magazines, books, and the internet.

### Features and Services

LifeCODE.ai and its synchronous online client DApp apply blockchain technology in an innovative manner to establish the legality and effectiveness of large-scale health data collection while protecting user privacy and creating data interaction value. As such, they truly realize the propertization and assetization of large health data. The main features of LifeCODE.ai are as follows:

- It ensures that health data are owned only by the individuals who upload their own health data and can never be changed without the owner’s acknowledgment.

- It establishes a trading mechanism for data exchange via tokens; these tokens are issued by the LifeCODE Databank, which was designed in accordance with Ethereum’s ERC-20 protocol, with a maximum issuance of 3,000,000,000 pieces. Medical and health institutions that wish to use health data must obtain the owners’ consent and pay a certain fee (ie, tokens) to *buy* the data. This positive mechanism can greatly encourage users to share their health data.

- The health data in LifeCODE.ai are stored on decentralized distributed nodes. All data and data transactions in the blockchain network are encrypted and traceable. The LifeCODE.ai platform also has a unique encryption algorithm and consensus mechanism that enable the resistance to tampering and traceability of health data, thus guaranteeing the privacy of the users’ health data.

LifeCODE.ai advocates blockchain as a service, which makes the use of blockchain as easy as surfing the internet. LifeCODE.ai mainly provides the following 3 product services:

- Smart contracts: It decouples decentralized smart contracts based on blockchain technology.

- Bookkeeping function: It uses distributed bookkeeping to address the lack of trust. Without a central organization, all parties have the same book to ensure that an open and transparent transaction process.

- Computational processing functions: It coordinates platform resources, processes gene sequencing data, and enables the data to be used.

### Platform Architecture

**Figure 2:**
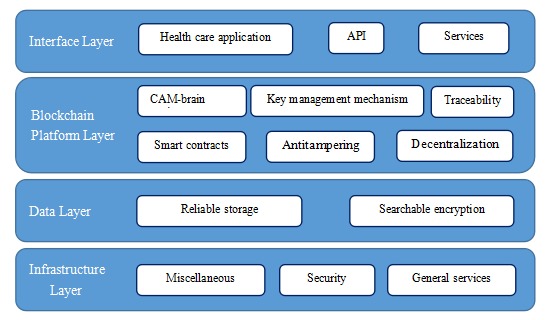
LifeCODE.ai platform architecture (adapted from LifeCODE.ai White Paper).

LifeCODE.ai integrates genomic data and phenotypic health data with the highest level of data quality and privacy to create a platform for data exchange, interoperability, and a wide range of services for all participants. The platform’s architecture is roughly composed of 4 layers (as shown in Figure 2): an infrastructure layer, a data layer, a blockchain layer, and an interface layer. The interface layer contains the application programming interface (API) and related business services for customers. The blockchain layer forms the platform’s core. In addition to traceability, antitampering, decentralization, smart contracts, and encryption algorithms, it also has a key management mechanism and a CAM-brain Engine. The data layer includes searchable encryption and trusted data storage that provide further protection for data security. The infrastructure layer contains basic services, among others, and a security module that comprises a network of access control, basic security service, and so forth. The blockchain layer, data layer, and infrastructure layer are used together to execute secure storage, complex algorithms, and other functions at the back end, thus providing stable services in the interface layer at the front end.

**Table 1:** Blockchain empowerment in genomics big data platform.

Empowered facets	Blockchain features	LifeCODE.ai solution	References
Data ownership	Traceability	LifeCODE.ai guarantees that the data belong only to individuals who upload their own health data. Only these individuals hold the private key for data management.	[12,13], [26,27]
Data sharing	Smart contracts, Antitampering	LifeCODE.ai establishes a set of conversion mechanisms between health data and assets. If platform participants wish to share or use data, they must follow the asset-based operating mechanism (ie, token mechanism).	[14,15], [28-31]
Data security	Decentralization, Encryption algorithm	LifeCODE.ai utilizes the features of blockchain technology to form privacy protection mechanism and a database with reliable storage, which guarantees the data security.	[16,17], [32-35]

### Case Analysis

The purpose of this study was to investigate how blockchain technology can empower the storage and management of genomics data in genomics big data platforms. The case evidence was analyzed across 3 facets as shown in Table 1: determining data ownership, establishing a data-sharing mechanism, and ensuring data security and privacy.

#### Determining Data Ownership

In LifeCODE.ai, the ownership of gene data absolutely belongs to the individual. Typically, once an individual uploads his or her personal genomic and phenotypic health data to the platform, the data are encrypted immediately and fully by default, and the business participants can access the data only with the owner’s permission. This model makes the platform just like a bank that stores and manages data. In the traditional case, although the gene data comprise individuals’ data, they were often managed by institutions such as hospitals. However, in the LifeCODE.ai blockchain, the rights are returned to the individuals, and each person’s data are stored on the platform and remain within his or her control such as a deposit in a bank. This decentralization of rights will give individuals peace of mind.

The technology to implement the determinacy of data ownership in individuals is blockchain. Specifically, in this process, the public and private key mechanism in the blockchain defines the data producer as the data owner and fully encrypts the data, thereby performing rights division and data protection. This private key mechanism allows data holders to securely store, access, and share their data via encryption keys under their own authorization. At the same time, traceability allows the blockchain to strictly monitor any access to the data and record it in LifeCODE blockchain to make all data transactions traceable. Thus, monitoring each processing record of the data is an operation authorized by the data owner, and the rights are locked in the individual’s hands, which will promote data self-governance. However, the autonomous nature of the blockchain plays down the requirement of state supervision. In the case that the supervision cannot be reached, some profit-seeking markets will lead the application of blockchain technology to the illegal field, thus causing certain risks.

#### Establishing a Data-Sharing Mechanism

**Figure 3:**
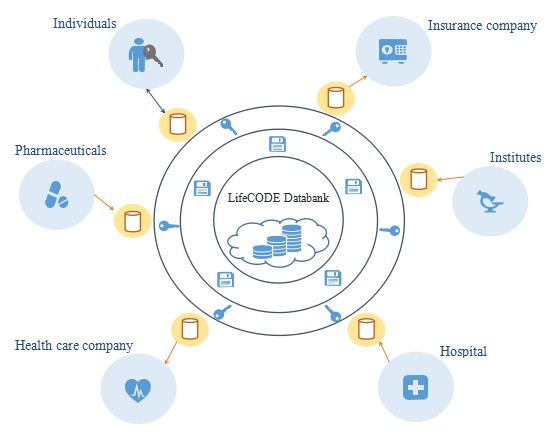
LifeCODE.ai operating mechanism.

LifeCODE.ai uses a token mechanism to enable data trading and sharing in a platform-based closed-loop ecosystem. Individuals, hospitals, research institutions, insurance companies, health care companies, and pharmaceutical companies are the major players in the LifeCODE ecosystem (as shown in Figure 3). The individuals are the data owners and hold the private key for data management, and other participants can communicate and trade with them to acquire or apply their data. For instance, if a research institution wishes to obtain 100 pieces of data from diabetes patients for drug R&D, they can search keywords such as *diabetes* in the platform. They can then obtain anonymous contact information of the relevant individuals, thereby initiating a data application and making them a token offer. The individuals can either approve the deal or reject it. The gained token through data transaction can be used by the individuals to obtain services from medical institutions and insurance institutions. This token exchange mechanism may enhance individuals’ positive motivations to share their personal genomics data.

To use the token mechanism to achieve data sharing, LifeCODE.ai not only uses the blockchain’s smart contract and antitampering features, it also effectively combines other technologies. In the process of data sharing, the smart contract code facilitates, verifies, and enforces the negotiation or performance of an agreement or transaction. The antitampering feature helps implement access and protect the originality of the data, thus ensuring the operation of smart contracts. The token mechanism involves 4 processes.

First, the data owner’s data are imported into the genomic ordered relational (GOR) architecture, which is a system specifically designed for genomic data storage, query, search, indexing, and many other analyses. The GOR architecture and the GOR pipe, the Clinical Sequence Analyzer (API), and Risk Engine API it provides can analyze the data to derive potential correlations between the genetic data and clinical phenotypes or diseases.

Second, according to the requirements, the data are fully or partially encrypted and imported into a searchable encryption database that was developed on the basis of asymmetric encryption algorithms, tag-based fingerprint extraction, and homomorphic encryption. Here, the data generate a corresponding label as an index to search by keyword.

Third, LifeCODE.ai aggregates, classifies, integrates, and indexes the phenotype data. As these data are so variable, LifeCODE.ai uses multiple approaches to manage and integrate them: (1) HL7-compatible specifications, currently under design, will be used to build general exchangeable and extensible data structures for both high-quality phenotype data and that from less-developed systems; (2) data from trusted sources verified by the permissioned LifeCODE blockchain are aggregated, indexed, and linked by logic-based and experience-based protocols and adapters; and (3) a master patient index mechanism is used to link an individual’s phenotypic data to an indexed keychain.

Finally, during the data transaction process, the secure transmission of data is the last layer of technical support for the sharing mechanism. The LifeCODE blockchain is a permissioned blockchain based on JP Morgan Quorum platform, an enterprise-focused version of Ethereum. Permissioned blockchain maintain an access control layer that allows certain actions to be performed only by certain identifiable participants. These blockchains differ from public and private blockchains.

However, the inability to tamper with the blockchain is both an advantage and a disadvantage. Due to the antitampering nature, once the incorrect account is transferred, it cannot be modified. The loss of the key will cause irreparable damage as well. In addition, transaction data will always be generated in the data-sharing project. As the body of transaction data continues to increase, the time required for the node to record the transaction data is also prolonged; the performance cannot be improved, and the efficiency will continue to decrease. Capacity will thus become a big problem for blockchain.

#### Ensuring Data Security and Privacy

The importance of genetic data security privacy cannot be overstated, so LifeCODE.ai adopts a combination of software and hardware based on blockchain to ensure data security. Data security and privacy can be summarized as 2 aspects: decentralized data storage and the data-sharing key mechanism. It is conceivable that reliable security can increase people’s sense of security or trust.

First, LifeCODE.ai guarantees the highest-level protection of data authenticity, privacy, and permanent data storage. All owner-authorized health data and associated data exchanges are securely recorded and stored to prevent data leakage, abuse, and loss. Specifically, LifeCODE.ai leverages the decentralization of the blockchain to offer reliable storage (object storage service, OSS/Hadoop Distributed File System, HDFS/network file system, NFS) of various types of health data in a secure cloud environment, matched with various forms of data-persistence implementations. OSS provides highly extensible and robust storage for genomic data. The HDFS is incorporated by LifeCODE.ai for high-performance storage, such as structured health data and complex mapping and correlation. The NFS and local storage are used by worker nodes to run analysis services and the API and to provide low-latency high-throughput data access.

Moreover, LifeCODE.ai exports the health data to an intermediate searchable-encrypted data repository for data analysis and research purposes, and only there can the data be searched. At the same time, the privacy-sensitive portion of the data is encrypted in the same way as the original data, and only data that cannot cause damage to the data owner can be searched. The LifeCODE.ai platform then cryptographically conceals the genomic assets of a transaction by applying *zero knowledge proofs,* which means that party A can prove to party B that he knows the specific information without revealing the information; thus, party A is the prover, and party B is the verifier. This feature is especially useful in cryptography because it provides an additional layer of privacy protection for the prover, and in the case of LifeCODE.ai, it protects the data owner. The LifeCODE.ai blockchain then supports private transactions through Cambrian, a peer-to-peer encrypted message exchange that secures data while communicating directly with network participants.

Nevertheless, this kind of security is not absolutely safe. The core of blockchain technology is cryptography, and its focus is the hash function. Many hash functions have been designed and widely used, but they generally have a short life span. Algorithms that are considered safe are often not successfully used for long periods of time, and new and more secure algorithms are designed successively. In addition, some imperfections remain in the computer languages upon which the blockchain relies. There will be a lag in the integration with new technologies that will affect the blockchain technology system. Unfortunately, the Ethereum blockchain has reportedly been hacked multiple times, resulting in the loss of currency.

## Results

### Blockchain as a Service

The blockchain-based genomics big data platform, LifeCODE.ai, is supposed to provide relatively safe and trustworthy data storage and management services to genomic stakeholders. As such, the platform can empower them to enhance precise medicine, personalized treatment, and new drug R&D. Specifically, it provides Blockchain as a Service, a new type of cloud service based on blockchain technology, for the genomic stakeholders including both institutions and individuals.

Even though LifeCODE.ai has been established less than a year, its tools have been successfully leveraged in several cases. For example, LifeCODE.ai provides blockchain data encryption and secure storage services to the National Center for Clinical Medical Research on Geriatric Diseases in China for storing and managing gene data of the patients with Parkinson and dyskinesia diseases. The research and analysis of this dataset can help to overcome the clinical research difficulties of Parkinson and dyskinesia diseases and promote the popularization and application of genetic testing technology in clinical diagnosis and treatment. However, LifeCODE.ai has not yet gathered enough users, and the limitations of the blockchain have not been well resolved. The future development of LifeCODE.ai still needs time verification.

**Figure 4:**
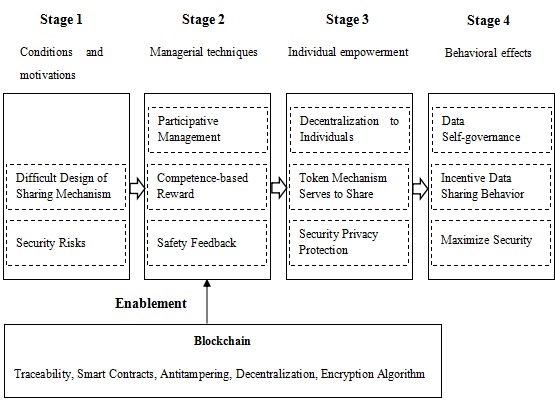
The empowerment process of blockchain for individual’s genomic data management.

To better understand the empowerment process of blockchain for individual’s genomic data exchange behavior, we draw on the research framework of psychological empowerment process. This theoretical framework was proposed by Conger and Kanungo (1988) to explain the process of empowerment in 5 stages including the psychological state of empowering experience as well as its antecedent conditions and behavioral consequences [46]. This model has been widely adopted to understand how to change the psychology of subordinates through empowerment behavior, thereby promoting behavior effects [47,48]. For example, this model was adopted to investigate how information system empowers users’ attitudes and promotes behavior effects [49]. On the basis of Conger and Kanungo’s empowerment process model and the case analysis of LifeCODE.ai, we proposed a framework to illustrate the empowerment process of blockchain for individual’s data exchange in a genomic big data platform. As there is no subordinate relationship in the big data platform, this study removed stage 3 of Conger and Kanungo’s framework. As can be seen in Figure 4, blockchain empowerment process can be divided into 4 stages as mentioned below.

### Diagnosis of Conditions and Motivations

As mentioned above, 3 obstacles hinder the process of establishing genomic big data platforms including unclear data ownership, difficult design of sharing mechanism, and security risk.

### Use of Managerial Techniques

Due to blockchain’s special features (ie, traceability, smart contracts, antitampering, decentralization, and encryption algorithm), individuals in the blockchain platform have the ability to participate in the data management process. They can also obtain token rewards from data transaction. The token rewards can be viewed as a kind of competence-based reward. Moreover, the application of blockchain technology can guarantee data security and privacy to a greater extent.

### Empowerment of Individuals

Blockchain technology provides new ideas for the establishment of a genomic big data platform. The application of blockchain technology assigns data ownership to the individuals and delegates power to them. The token mechanism can serve the data-sharing process, and the security and privacy of the data are better protected.

### Leading to Behavioral Consequences

Ultimately, the above aspects will result in changes in the behavior of the individuals in the platform. Personal ownership of data will facilitate the autonomy of personal data. The token mechanism will encourage more active sharing between individuals and institutions. Besides, reliable protection of security and privacy will encourage individuals to use this platform more actively.

## Discussion

### Conclusions and Implications

We conducted a case analysis to show how blockchain can empower the storage and management of personal genomics data from the perspectives of data ownership, the data-sharing mechanism design, and data security. We found that blockchain can allocate data ownership to individuals because of its traceability feature, so that individuals can control and monitor all the activities of their genomic data. This process leads to decentralization of data ownership and facilitates the autonomy of personal data. Meanwhile, the blockchain can use its smart contract and antitampering features to design a token-sharing mechanism promoting more active data sharing between individuals and organizations. Last but not least, the distributed storage and secret key mechanism, resulting from blockchain’s decentralization feature and cryptographic feature, respectively, provide greater guarantee for the security and privacy of the genetic data. This security feedback will increase confidence in the personal application platform.

In conclusion, the blockchain platform provides new possibilities for the management and security of genetic data and can help realize the psychological empowerment of individuals in the process, and consequently, the effects of data self-governance, incentive-sharing, and security improvement can be achieved. As concepts of data ownership vary among cultures and laws vary around the world, it is worth noting that the question regarding data ownership needs further exploration. The discussion in this paper is based on the Chinese background, so it is more reasonable to hand over the ownership of data to the data producer (which is individuals) without the specific guidance of the law. Undeniably, the blockchain is far from perfect. Regulatory issues, capacity and efficiency issues, and hash cryptographic security issues remain challenges in the current phase of blockchain.

### Limitations and Directions for Future Research

This study has several limitations that call for additional research. First, we selected only 1 blockchain-based genomics big data platform in China (ie, LifeCODE.ai) as our research object. A future study should compare LifeCODE.ai with similar platforms to enhance generalizability. Second, because LifeCODE.ai has been in use for less than a year, successful cases are still limited. Its client DApp (Lai-Tribe) has only around 5000 registered users. It takes time to prove whether users and medical institutions will be willing to store and share their genomics and health data in this blockchain-based platform. Future studies are needed to investigate the attitudes of individual users and medical institutions toward blockchain-based data storage and management services provided by LifeCODE.ai and similar entities. Third, at the current stage of blockchain technology, some problems remain to be solved, such as capacity issues and regulatory issues. Therefore, the study of blockchain in gene data management and security assurance requires long-term follow-up and in-depth research with multiple cases and multiple angles.

## Abbreviations

API: application programming interface
DApp: decentralized app
GOR: genomic ordered relational
HDFS: Hadoop Distributed File System
NFS: network file system
OSS: object storage service
R&D: research and development
